# Improving financial protection and access: Managing co-payments for outpatient prescription medicines through digital technologies in Estonia^☆^

**DOI:** 10.1016/j.hpopen.2026.100176

**Published:** 2026-06-30

**Authors:** Kaija Kasekamp, Triin Habicht, Inke Mathauer

**Affiliations:** a Barcelona Office for Health Systems Financing, WHO Regional Office for Europe, Sant Antoni Maria Claret, 16708025 Barcelona, Spain; b WHO headquarters, Department of Performance, Financing and Delivery, Avenue Appia, 1211 Geneva, Switzerland

**Keywords:** Digital technologies, Financial protection, Outpatient prescription medicines, Co-payment management

## Abstract

**Introduction:**

Estonian households faced persistent financial hardship due to high out-of-pocket payments for outpatient prescription medicines. An “additional medicine benefit” was therefore introduced in 2003, which lowers co-payments once a person's spending for outpatient prescription medicine has reached a threshold. However uptake remained low due to administrative hurdles and lack of awareness. In response, Estonia reformed this benefit in 2018, including leveraging digital technologies to automate eligibility checks and benefit provision at the point of sale. This paper explores how Estonia developed, designed and implemented these digital technologies and analyses the reform's observed changes.

**Methods:**

This study employed a qualitative-dominant mixed-methods approach, including document reviews, key informant interviews, and analysis of administrative data from the Estonian Health Insurance Fund (EHIF). The WHO framework for assessing digital technologies in health financing guided data collection and thematic analysis.

**Results:**

Built on Estonia's e-prescription system, the reform included lowering co-payment thresholds coupled with automating a member's benefit and enabling related real-time data exchange. The share of patients benefitting rose from 0.4% in 2017 to 15.6% in 2018, which is assumed to have contributed to decreasing the incidence of catastrophic health spending during the same time. Automation has significantly reduced the administrative burdens for EHIF, pharmacists and patients.

**Conclusion:**

Estonia's experience demonstrates how digital technologies support health financing reforms, contributing to increased efficiency, improved financial protection and utilization aligned with need. Yet, there is further potential to use existing data and digital technologies to better target benefits to those most in need.

## Highlights

• The paper analyses Estonia's additional medicine benefit reform and its use of digital technologies.
• The use of digital technologies supports progress towards universal health coverage objectives.
• Automation and real-time data exchange are key digital elements to realize the reform's objective.

## 1 Introduction

Out-of-pocket (OOP) expenditure and co-payments for health care including (prescription) medicines pose a huge problem in many countries of all income-groups, as they affect financial access and financial protection, in particular for lower-income households or those with chronic disease [1]. Bringing down co-payments is thus an important objective, requiring changes in health financing policy and adjacent areas (WHO, 2020). One policy option is to exempt targeted population groups from co-payments or to reduce these, but the targeting precision and financial protection effect remains limited in many countries across income levels [[2], [3], [4]].

Growing evidence across regions and countries of different income levels suggests that various digital technologies can play an important role in supporting various health financing functions [5]. One such area is the digitally supported provision of (additional) service or medicines benefits - for instance through real-time data management and automation - by managing and limiting high OOP expenditure to enhance financial protection. Studies from the United States reveal strong interest in and expressed need for digital technologies by healthcare providers to help them bring down OOP expenditure on prescription medicines for their patients. These studies also point to the potential of digital tools for real-time benefits and price transparency to help patients manage cost-sharing [[6], [7], [8]]. In practice, various OECD and high-income countries, such as Australia, Austria, Finland, France, Belgium, Japan, Norway, Spain and Sweden [[9], [10], [11], [12]], have set OOP expenditure thresholds, after which additional medicines costs are fully or partially covered, through an automated process, for all or selected population groups. Likewise, Estonia reformed its additional medicines benefit and introduced an automated provision of it in 2018 through digital tools.

However, we are not aware of any studies that document and assess these digitalized processes, at least not in English language, pointing to a gap in the literature on how digital technologies are designed and implemented to support the realization of coverage policies with the objective of enhanced financial protection. Yet, with the digital transformation in the health sector taking place in countries across all income levels, countries seek to understand how to develop, design and implement digital technologies for this purpose.

This paper closes this gap by examining how the use of digital technologies in Estonia served to realize the coverage policy reform, namely the revision and expansion of the additional medicines benefit with the goal of enhancing affordable access to these outpatient medicines. First, this paper explores how such digital technologies were in practice developed, designed and implemented in Estonia. Second, it explores the observed changes after this reform. The insights from Estonia may provide lessons to policy makers in other countries who seek to use digital technologies to realize the implementation of their health financing policy reforms. The remainder of this Introduction outlines Estonia's situation prior to its 2018 reform of the additional medicines benefit.

Estonia is a small country with a population of 1.37 million [13], with an efficient public service supported by advanced use of digital technologies and characterized by high digital literacy among the population [14]. In the health sector, the digitization of health care data began in the 1990, led by the country's single purchaser, the Estonian Health Insurance Fund (EHIF) [15].

Estonia has been equally struggling with high OOPs and a complex co-payment arrangement for prescription medicines [[16], [17]]. A reimbursement system for outpatient prescription medicines for the population covered by the Estonian Health Insurance Fund (EHIF) has been in place since 1993. In 2003, two levels of annual aggregate spending thresholds per person for prescription medicines were introduced, at which higher co-payment reductions kicked in. This was called the ˝additional medicines benefit˝ (in the following referred to as ˝the benefit˝) [[18], [19]]. Importantly, these thresholds did not constitute caps for patients, but the benefit aimed to reduce the financial burden for patients with high needs for prescription medicines. This benefit was retroactively reimbursed (in cash) after the patient submitted an application. The threshold amounts related to percentage co-payments were decreased several times over the following years. Yet, many eligible patients did not apply, as they did not know about being eligible and as it was cumbersome, thus foregoing the benefit [20].

As a result, OOP payments for prescription medicines remained one of the most significant categories of OOP spending in Estonia, accounting for 18% in 2017 [21]. Based on 2016 data, the incidence of catastrophic spending on health was higher in Estonia than in many European Union countries, and 5.54% of households were impoverished, further impoverished or at risk of impoverishment after OOP payments. Among households in the lowest income group, outpatient medicines emerged as the primary cause of financial hardship [16]. The Ministry of Social Affairs (in charge of health) initiated a reform to address this problem, whereby digital technology played a decisive role to materialize this reform.

## 2 Materials/methods

### 2.1 Conceptual framework

Digital technologies can be defined as electronic tools, systems, and devices, that generate, store, use and analyse data [22]. If well designed and implemented, digital technologies may contribute to progress towards the Universal Health Coverage (UHC) goals: utilization in line with need, financial protection and quality of care, often specifically addressing the intermediate UHC objectives of equitable distribution of resources, efficiency, transparency [[5], [22], [23]]. This paper is looking specifically at benefits design and condition of access to health services, i.e. co-payment rules.

The study was oriented by the World Health Organization's (WHO) guide to assess the effects of digital technologies on health financing and UHC objectives [23]. Specifically, our theory of change, as depicted in the box headings of Fig. 3, assumes that policy changes in the coverage policy, combined with the introduction of various digital elements, change and simplify administrative processes for both pharmacists as well as patients. This is expected to improve access to the medicine benefits. Utlimately, this is assumed to contribute to the final UHC goals of utilization in line with need (less foregone medicine purchases) and improved financial protection (lower incidence of catastrophic expenditure), although our observational approach cannot confirm or measures this, more so due to other confounding factors.

### 2.2 Data collection and analysis

This study used a qualitative-dominant mixed-methods approach [24] to document and assess the evidence and context around which digital technologies were designed and implemented in Estonia and the results it produced.

At the start, a review of available documents was undertaken, including published peer-reviewed and grey literature, government policies, implementation and evaluation reports, and other relevant documents. This was followed by holding in-depth interviews. The document review also informed the development of the interview guide as well as the sampling strategy.

The key themes included in the interview guide focused on how the digital technologies were used for the provision of the benefit and how the digital technologies benefited EHIF's purchasing function and potentially contributed to UHC objectives. The interview guide was iteratively refined within the research team to ensure clarity, relevance and alignment with study objectives.

A purposive expert sampling strategy was employed to identify individuals with direct, in-depth knowledge of the design and implementation of the reform of the additional medicines benefit in Estonia. Selection criteria included: direct involvement in policy implementation, technical expertise in health information systems or digital solution development related to medicines coverage, and active engagement in decision-making or operational processes during the reform period. The final sample comprised four key informants (2 women, 2 men), which reflects the limited pool of experts nationally engaged in this highly specialised policy area.

Two of these interviews were conducted online using MS Teams and two interviews were done in person. Interviews were semi-structured, allowing focus to vary, depending on the knowledge of the expert. During interviews, notes were taken. Across the interviews, a high degree of convergence in responses was observed.

A thematic analysis of interview notes was conducted by authors: deductive codes were based on the WHO guide [23], whereas inductive codes also emerged from the data, with themes interpreted and synthesized to understand the design and implementation process, to explore the changes assumingly resulting from the digital technology, as well as to identify challenges. Coding and theme development were discussed within the research team to ensure consistency and coherence. Researcher triangulation and iterative discussion were used to validate interpretations and ensure credibility of the findings.

Finally, descriptive statistical analyses were conducted to examine trends over time, including pre- and post-reform comparisons. We collected administrative data from EHIF from the period 2017–2025, enabling comparison of indicators before and after key policy changes. Changes in benefit coverage and uptake were assessed using annual aggregate data. The data quality and coverage are assumed to be high as it is directly linked to providing benefits to the entire population and the EHIF dataset covers 100% of the insured population) [19]. Data on the incidence of catastrophic health expenditure were obtained from the WHO UHC Watch database ([16]. These data were analysed descriptively alongside EHIF indicators to describe broader system-level trends.

### 2.3 Ethics and consent to participate

As findings are based on document review and on the interviews of experts working in their core area of expertise, no ethical approval was sought for this work. The policy experts are not considered research participants in the conventional sense as they are not the subject of the research, but rather are sharing information on it. Hence, any ethical risks to them are unlikely to arise [25]. Experts gave informed consent to participate. We have anonymised the content of expert interviews.

## 3 Results

### 3.1 The starting point: The introduction of the e-prescription system and the additional medicine benefit

The health system's key digital technologies that have provided the foundation for and enabled the reform of the additional medicines benefit include the national identity card system with a unique personal identifier, authentication mechanisms, the digital claims management system of the EHIF, and the nationwide digital health information system. The latter led to the centralization of data of different healthcare providers and also allowed the exchange of data between them [26].

The digital claims management system set up in the early 2000s is a uniform, centralized and secure digital data exchange service, called electronic channel (abbreviated as e-channel) to transmit data between contracted health service providers and EHIF. As of October 2002, all pharmacies were obliged by law to digitally transmit prescription data to EHIF. Physicians issued paper-based prescriptions, which pharmacists then digitalized by entering them into their information management systems in order to claim reimbursement by EHIF. This made the process less time- and resource-consuming [15]:

“The discussion on introducing an additional medicines benefit emerged once an evidence base from data became available. [...] The system had to be built so that decisions could be made based on data” *(*key informant 2).

Building on the above, during 2008–2010, the EHIF designed and piloted an e-prescription system. Healthcare providers and pharmacies were advised to align their information systems with the e-prescription service over time. By 2010, when the e-prescription system was officially launched, pharmacies were legally mandated to process electronic prescriptions through the e-prescription service (key informant 2).

The e-prescription system consists of the e-prescription service to enable data exchange, and the e-prescription database, a central data repository to store the data and to communicate with other relevant databases. The service is built to be needs-based: when a physician issues a new prescription using the e-prescription service, it is sent to the e-prescription database, and the relevant information can be retrieved from the e-prescription service by the physician (to review the data on previous e-prescriptions), by the patient (to review their e-prescription in the Eesti.ee portal) as well as by the pharmacist (to fill the prescription) [20].

The e-prescription database is the core of the system, connecting with EHIF's data systems and public registries to verify the issued prescription in real time, including the provider's prescribing rights, the pharmacist's dispensing rights, and the validity of prescribed medications. EHIF's own data systems serve to check the list of reimbursable medicines, medicine reference prices and entitlement rules, all of which are updated regularly. The e-prescription database retrieves these data in real time, while data with other public registries is synchronized once a day (key informant 3). The e-prescription system represents a critical digital infrastructure that laid the foundation for the subsequent reform steps outlined below.

The additional medicine benefit was introduced in 2003. Table 1 (column 2) provides a summary of the benefit design features as of 2003. EHIF used digitized prescription data to calculate a patient's (up to that point aggregate) co-payment expenditure and the related benefit, so that a patient did not need to retain their original prescriptions (key informant 2). Nonetheless, the benefit was not used as envisaged because of various hurdles. First, despite public information to patients and physicians receiving training to inform their patients, many people remained unaware of the benefit or struggled to estimate their eligibility (key informant 2). Second, they had to submit an application to EHIF to receive the benefit, but many did not. The application could be in any format (email, letter or in-person) that expressed the person's intent to receive the benefit, whilst providing their ID code and bank account information. Based on the claim submissions received by pharmacists, EHIF assessed the patients' eligibility at the end of each quarter, providing payments to those who had applied at least once in their lifetime and met the qualifying criteria. As a result, many eligible people did not apply.

**Table 1:** Key design features of the additional medicines benefit when introduced in 2003 and after the reform in 2018.

Outpatient prescription medicines		2003	2018
Fixed co-payment amount		1.27 EUR for medicines for specific conditions, children, people receiving a state pension or with partial or no capacity to work, or for medicines to treat severe or life-threatening conditions • 3.19 EUR for all other beneficiaries and medicines	Same for all medicines: 2.50 EUR

Percentage co-payments depending on the medicine and age group:		• Standard prescription medicines: 50% • Medicines for specific conditions: 25% • Medicines for specific conditions, for children aged 4–16 years, people receiving a state pension, aged over 63 years or with partial or no capacity to work: 10% • Medicines for children aged under 4 years and for all insured for severe or life-threatening conditions and epidemics: 0%	The same rates apply

Additional medicine benefit applied to reduce co-payments for patients with high co-payment levels	Threshold amount per calendar year above which the additional medicine benefit reduces co-payment of 50%	384 EUR	100 EUR

	Threshold amount per calendar year above which the additional medicines benefit reduces co-payment of 90%	639 EUR	300 EUR

Threshold calculation		Threshold includes only percentage co-payments	Threshold includes both percentage co-payments and fixed co-payments

Beneficiary's application required to get the benefit		Yes	No

Beneficiary needs to know whether eligible		Yes	No

EHIF granting the benefit		Retrospective reimbursement, based on an application	Automatic provision, at the point of buying the medicine

Source: Authors' compilation (adapted and expanded based on [20]).

A subsequent improvement was the digitalization of the application process, allowing patients to submit their application for reimbursement of parts of their medicine expenditure via the Eesti.ee portal or the Patient Portal, introduced in 2008. This further simplified the steps to undertake, yet for people with limited mobility or no internet access, applying remained difficult. Nonetheless, people still had to pay co-payments upfront, apply and wait for the retrospective reimbursement of co-payments, potentially discouraging use. These hurdles may also have led to an unknown number of people with unmet need as patients may have decided not to buy the medicines in the first place. Moreover, even though the co-payment thresholds had been lowered several times during the period of 2003–2018 to enlarge coverage, data on OOP payments and financial protection of households continued to indicate significant financial burden caused by prescription medicines [20].

### 3.2 The decisive changes in coverage policy and digital technology: Design and implementation

It became evident that if the Government of Estonia wanted to improve financial access to medicines and strengthen financial protection, there was a clear need to change the way of accessing the benefit to ensure that all eligible people could benefit with the help of digital technology.

Thus, in spring 2017, the government decided to further reform the additional medicines benefit to improve financial protection and increase provision of the beneft with one key element being the shift from application-based to automatic provision of the benefit, supported by strong commitment from pharmacies (key informant 1):

“Pharmacists were interested in the reform as it made the medicines more accessible. The technological developments required on the pharmacy side were minimal, and thus they themselves absorbed these costs” (key informant 1).

In 2018, the reformed benefit came into place, based on two major changes. First, the threshold levels for the additional medicine benefit to kick in were again lowered. As a result, more patients would thus reach the threshold and become eligible. Second, the provision of benefit shifted from a retrospective cash benefit to a real-time in-kind benefit that people would receive at the point of buying a medicine in a pharmacy, abolishing the application process, thus ensuring that all entitled patients would receive the benefit right away (see Table 1).

**Fig. 1:**
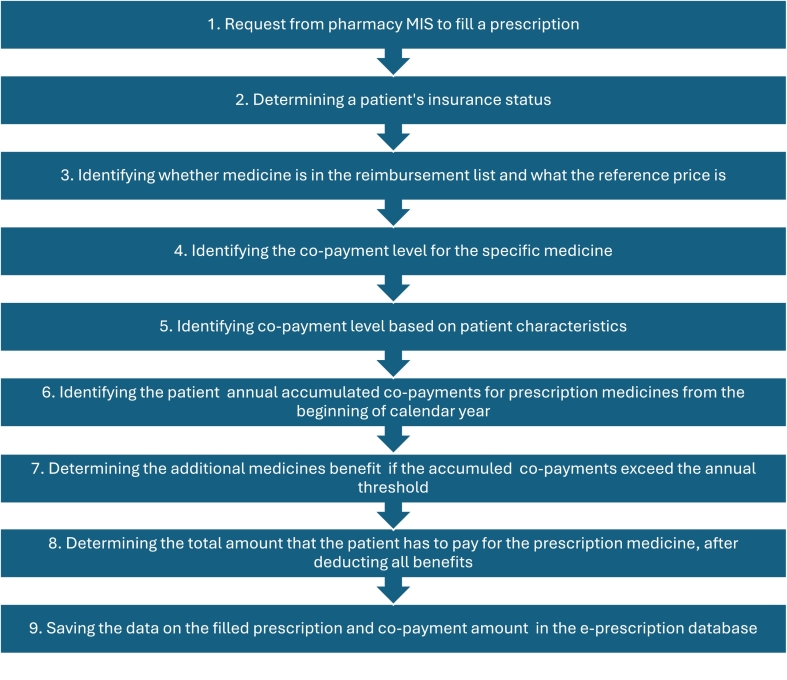
Steps to determine the co-payment amount for a prescription. Source: Authors' compilation (adapted from [20]).

All the necessary data for determining patient eligibility and calculating the benefit was already available in the e-prescription system. Hence, only a few but critical changes to the e-prescription database and service were required, namely the inclusion of a specific algorithm in the existing e-prescription service to automate the calculation of the additional benefit and enabling real-time data exchange with pharmacies on a patient's accumulated co-payments. Fig. 1 outlines the entire digital automation process. With these digital changes, the benefit could be provided automatically and immediately, thus reducing co-payments at the time of buying the medicine.

People no longer need to be aware of the benefit, check whether they are eligible or make an application to receive the additional benefit through retrospective reimbursement:

“The fundamental decision was that the digital technology would allow automatic application of the benefit in the pharmacy which would ensure that the benefit applies to everyone who is eligible, and at the pharmacy the person cannot say that they do not want it” (key informante 1).

**Fig. 2:**
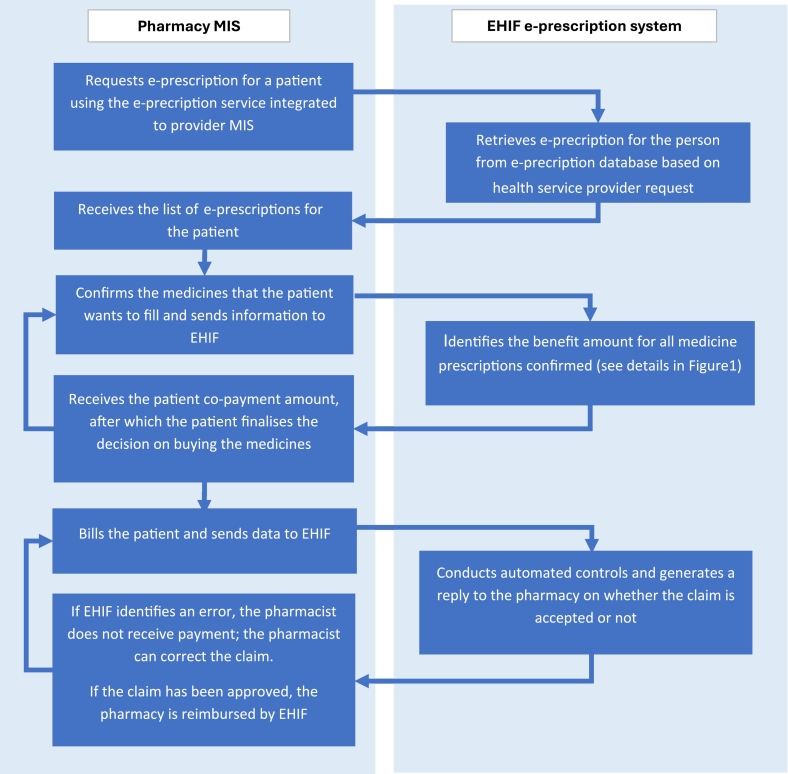
Pharmacy MIS steps and data exchange with EHIF for the sale of a prescription medicine. Source: Authors' compilation (adapted from [20]).

The steps and API-based (Application Programme Interface) data exchange processes for a pharmacy management information system (MIS) are described in Fig. 2. At the start, the pharmacist identifies the person based on the ID code, allowing the pharmacist to retrieve patient data related to the prescription. After confirming the medicines the patient wants to purchase, the pharmacy MIS sends this information to the e-prescription database, which then calculates the patient's co-payment. This “confirming” step is critical, as it triggers the benefit calculation. At this point, the pharmacist can inform the patient of the co-payment amount and whether they have reached the threshold for the additional medicine benefit (key informant 3).

The first stage of the software development and its initial implementation was led by HIF. A private company, already familiar with the e-prescription system being a long-term EHIF partner, carried out the outsourced work. The cost of the development was approximately €50,000. The internal work conducted by EHIF information technology specialists, including maintenance and monitoring, was not separately budgeted, as it was considered part of their routine responsibilities. Nevertheless, it is important to note that maintaining such sophisticated IT systems requires a strong IT capacity within the EHIF employed staff. Moreover, EHIF worked closely with pharmacies to update their pharmacy MIS (key informant 3).

### 3.3 Observed changes after the introduction of the reform and the use of digital technology

The data suggests that the reform made medicines more affordable for a much larger number of people (see Table 2). On the one hand, we observe that the decrease of the thresholds of co-payments for medicines per calendar year is followed by a significant increase in the number of people entitled to the benefit from this protection mechanism. On the other hand, introducing automation of the calculation process and enabling real-time exchange of this data as part of the digital process meant that 100% of eligible patients benefit.

**Table 2:** Observed changes in relation to the additional medicine benefit expansion and its automation.

**Indicator**	**2017** **(before the reform)**	**2018** **(after the reform)**	**2019**	**2020**	**2021**	**2022**	**2023**	**2024**	**2025**
**After lowering the annual spending threshold from €300 to €100:**									
Number of people eligible for reduced co-payments⁎	8000	134,000	136,000	141,000	146,000	154,000	165,000	177,000	225,000

**After automating protection from user charges:**									
Share of eligible people benefiting from reduced co-payments	37.5%	100%	100%	100%	100%	100%	100%	100%	100%

**Changes after the lowered threshold combined with introducing automation:**									
Share of patients filling a prescription benefiting from reduced co-payments	0.4%	15.6%	15.8%	16.9%	17.3%	17.4%	18.4%	19.5%	24.8%

Source: EHIF [27], WHO [17]

⁎ The numbers are rounded to thousand units.

Over this period, the UHC Watch data shows that the incidence of catastrophic health spending decreased overall by 11% (8.1% in 2016 versus 7.2% in 2019) and in particular in the poorest quintile by 20% (4.6% in 2016 versus 3.7% in 2019) [16]. This suggests that the additional medicines benefit reform has contributed to improved financial protection, although there are also other contributing factors. For instance, increases in employment and living standards may have strengthened households' ability to pay for health care, which grew faster than the cost of meeting basic needs between 2015 and 2020 [28].

**Fig. 3:**
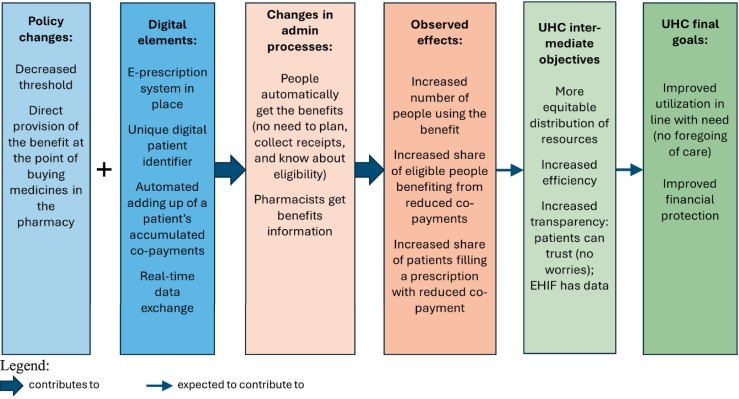
Changes in the additional medicine benefit and digital elements, changes in processes and observed effects. Source: authors.

The reform also reduced the workload of EHIF's Customer Support and Finance Departments. The former no longer handles patient applications for the additional medicines benefit. Moreover, the Finance Department no longer has to process cash transfers. However, since relatively few people had received the benefit before the reform and as the internal calculation process had already been automated via the e-prescription system previously, the reduction in EHIF's administrative costs was minimal during that final step (key informant 3). More importantly, the new process significantly eased the burden on individuals, who no longer needed to apply – though this impact is harder to quantify. Fig. 3 provides a visualization of the observed results chain.

## 4 Discussion

### 4.1 The potential of digital technology to support health financing policy and universal health coverage objectives – Lessons for other countries

This is the first study of its kind sheding light on how digital technologies are used for providing and automating prescription medicines benefits, thus removing administrative barriers and increasing people's access to the additional medical benefits. The policy revisions between 2003 and 2018 were insufficient and revealed that improving coverage required the embracing of digital solutions to realize the policy effectively to have actual impact. In other words, this improved coverage policy could not have been brought to fruition without the automated eligibility checks and automated calculation processes for providing the benefit. The efficiency gains from automation and use of real-time data have also been revealed elsewhere, including in low- and middle-income countries [5].

The Estonian experience illuminates the potential of the various digital elements to contribute to UHC objectives: The e-prescription system and the availability of digital data constituted the enabling digital public infrastructure and starting point for the subsequent additional medicine benefits reform. This enabled to introduce less bureaucratic and burdensome processes of managing patient benefits for both people and the health administration. The digital data include each person's unique ID, which allows patient tracking and this proved to be decisive to automatically determine and calculate whether a person is eligible for a benefit. Altogether, the shift from an application-based process to a fully automated system in 2018 reduced the administrative burden for EHIF and pharmacists, but foremost for patients. Streamlining business processes resulted in cost savings for all actors. Digitalization and automation are thus strong contributing factors towards the intermediate UHC objective of efficiency. A number of middle-income countries have set up digital claims management systems [5], which generate data. This could be used for predictive analysis [29] and hence for estimating co-payments and triggering exemption for patients with high healthcare needs.

As the resources in a healthcare system are limited, it is crucial to strategically select benefits and services and channel them to population groups most in need, i.e. people with high expenditure on prescription medicines, such as low-income households or the chronic sick. Digital data has made it possible in Estonia to make the distribution of resources more equitable by providing additional benefits to those population groups with higher OOP payments for outpatient prescription medicines. The availability of digital data linked to patient characteristics is key, and various machine learning modelling exercises have equally shown in other countries that claims and medical data can be used to reallocate resources to those most in need, with lower co-payments as a consequence ([30], for Pakistan), or to predict OOP expenditure of households ([31], for Türkiye).

A third key element, based on the availability of digital data, is the automaton of granting an in-kind benefit to all those eligible at the point of needing and buying a medicine. Low-income households in particular may not have the resources to pay co-payments upfront and hence benefit most from replacing a retrospective cash benefit by an in-kind benefit applied at the point of using the service.

An interesting point to note is that the EHIF co-payment rules are still complex, including different rates of percentage co-payments for different services and different population groups. This is difficult for a lay person to understand. However, with the digitalized and automated system, there is no longer need for an individual patient to be on top of these rules and people can and do trust the government agencies. Nonetheless, the digitalization brings additional transparency and clarity to patients, as they are given all information on their ills and accumulated co-payments in the Eesti.ee patient portal.

It is equally important to point out that automation did not require complex developments in information technology; it basically meant the addition of some algorithms. Nevertheless, it is not self-evident and is the expression of an explicit choice. Automation implied a change in mindset of EHIF: getting the additional benefits turned into a right to which people are entitled and that they should automatically get, rather than claim for it. In fact, very few European countries have introduced automation processes within their coverage policy implementation to provide income-based caps on co-payments [12]. There are also promising entry points for middle- and low-income countries: Digital wallets for health care in Kenya and Rwanda [32] and elsewhere [33] and mobile-phone applications, such as Indonesia's Mobile JKN [34], would have the potential for automated co-payment tracking and could be adapted to trigger exemptions once spending exceeds a defined threshold.

### 4.2 Remaining challenges and policy options

Despite a major reform and the use of digital technologies, financial protection remains a challenge in Estonia. As per 2020 data, many households still face high OOP payments, and the share of households experiencing catastrophic health spending (7%) is still higher than the EU median (4%) [[16], [28]]. Outpatient medicines remain a key cause for financial hardship, particularly for low-income households. Continuous monitoring and adaptive policy adjustments, for instance additional protective measures for those in greatest need are hence essential.

Yet, the existing data is not used to its full potential to identify those most in need. Better protection could focus on households at high risk of financial hardship. Reducing OOP payments for outpatient medicines and expanding benefits for low-income groups would decrease both unmet need and financial hardship. Estonia could also consider introducing a cap on total co-payments, using EHIF's digital systems to track a person's accumulated costs. This would allow for an automatic ceiling on co-payments, tailored to income, without requiring any application. Moreover, the thresholds could be applied on a monthly rather than an annual calendar basis, thus helping provide regular support and improve access for those on lower incomes. This adjustment would be simple to implement using existing digital systems. Non-exploited data is equally an issue elsewhere. The challenge does not seem to be the technical aspect, nor the data availability, but data governance issues.

### 4.3 Study strengths and limitations

It is important to note the difficulty in attributing improvements in access to medicines to the changes in digital technology alone, as the digital technology supports a policy reform that itself has also effects on the UHC objectives. Other contextual and potentially confounding factors could have equally played a role. Moreover, there were continuous adjustments to the medicine benefit package itself (new medicines added, changing percentage co-payments for certain medicines) as well as shifts in medicine price levels, all of which affected the level of co-payments and hence the observed changes. Our analysis was descriptive in nature, i.e. results should not be interpreted as causal effects. Nonetheless, the overall direction of the change is still valid, as demonstrated by the findings.

Another challenge is that much of the reform process (e.g. rationale for decisions) on implementation related details was not systematically documented at the time. Part of the data was collected through retrospective interviews with some risk of cognitive bias, but triangulation helped to minimize this risk. The small sample size may still limit the diversity of perspectives captured. The study did not capture perspectives from healthcare providers or other implementers for a deeper understanding of operational challenges and frontline experiences.

## 5 Conclusion

This paper has explored the use of digital technologies to realize a reform of the additional medicines benefit in Estonia. Specifically, it examined how web services and software supporting inter-operability and real-time data exchange between the EHIF, Ministry, doctors and pharmacies as well as automation were decisive for this reform to be put in practice. The redesigned digital system automatically tracks co-payments on prescription medicines and provides real-time benefits once an annual threshold is reached, eliminating the need for applications. This reform significantly increased the share of people benefiting from reduced co-payments, and can thus be considered as one contributing factor for reduced catastrophic health spending, with the largest decrease as per the UHC Watch data among the poorest quintile.

The Estonian experience shows that affordability of prescription medicines can be improved through various digital elements. This involved digitizing data and using unique patient identifiers to target those who are most in need and digital technology that enabled the automatic provision of benefits to everyone eligible at the point of use. Altogether, this contributed to the final UHC goals of financial protection and utilization in line with need (i.e., people in need for medicines are able to afford and purchase them), although the precise contribution is difficult to establish in view of other factors. While Estonia is known for its advanced digital system and data availability, the IT changes to accomplish this policy were not overly complex. The Estonian experience provides achievable lessons for other countries, particularly low- and middle-income countries seeking to bring down co-payments notably for vulnerable population groups.

## Credit authorship contribution statement

**Kaija Kasekamp:** Writing – review & editing, Writing – original draft, Visualization, Validation, Methodology, Formal analysis, Data curation, Conceptualization. **Triin Habicht:** Writing – review & editing, Writing – original draft, Validation, Project administration, Methodology, Formal analysis, Conceptualization. **Inke Mathauer:** Writing – review & editing, Writing – original draft, Visualization, Project administration, Methodology, Funding acquisition, Formal analysis, Conceptualization.

## Funding

The work on this manuscript was part of a larger study, for which we gratefully acknowledge financial support received from the Government of Canada.

## Declaration of competing interest

The authors declare that they have no known competing financial interests or personal relationships that could have appeared to influence the work reported in this paper.
